# Materialism, Egocentrism and Delinquent Behavior in Chinese Adolescents in Mainland China: A Short-Term Longitudinal Study

**DOI:** 10.3390/ijerph19084912

**Published:** 2022-04-18

**Authors:** Daniel T. L. Shek, Diya Dou, Xiaoqin Zhu, Xiang Li, Lindan Tan

**Affiliations:** Department of Applied Social Sciences, The Hong Kong Polytechnic University, Hong Kong SAR 999077, China; diya.dou@polyu.edu.hk (D.D.); xiaoqin.zhu@polyu.edu.hk (X.Z.); xann.li@polyu.edu.hk (X.L.); lindan.tan@polyu.edu.hk (L.T.)

**Keywords:** adolescent, materialism, delinquent, egocentrism, longitudinal, replication, Chinese students

## Abstract

Although research generally showed that holding materialistic beliefs would lead to poor developmental outcomes, few studies have used adolescent delinquency as an outcome measure. In addition, the intervening processes between materialism and adolescent developmental outcomes are unclear. In particular, it is not clear how materialistic beliefs influence egocentrism and adolescent delinquency. Methodologically, the existing studies have several weaknesses, including small samples, cross-sectional research designs, and being limited to people living in Western cultures. Using two waves of data collected from Sichuan, China (N = 4981), we studied the predictive effect of adolescent materialism on delinquency and the mediating role of egocentrism. Over two occasions separated by six months, students aged 11 and above responded to a questionnaire evaluating adolescent materialism, egocentrism, and delinquency (mean Wave 1 age = 13.15, range between 11 and 20.38). Results of multiple regression analyses suggested that materialism at Time 1 positively predicted Time 2 egocentrism. Additionally, Time 1 materialism positively predicted the level and change in Time 2 delinquency. Finally, based on 5000 bootstrap samples with gender, age, ethnic group, and Time 1 delinquent behavior as covariates, PROCESS analyses showed that egocentrism partially mediated the influence of Time 1 materialism delinquency and its change at Time 2. This study suggests that materialistic beliefs shape egocentrism, which further strengthens adolescent delinquent behavior. This study also replicates the findings of a pioneer study in China reported previously.

## 1. Introduction

For adolescents, the desire to possess trendy commodities may contribute to delinquent behavior such as stealing. In a report on knife crime in London, Lais [1] used the headline “children lured into crime because of materialism”. In the scientific literature, researchers have used the concept of “materialism” to understand materialistic behavior, which commonly refers to preoccupation with material possession such as money and luxury items. In the early work on materialism, Belk [2] proposed “possessiveness”, “non-generosity”, and “envy” as defining attributes of materialism. Richins and Dawson [3] also pointed out that different conceptions of materialism share the following elements: acquisition of materials plays a central role in one’s life, and success and happiness are defined in terms of material possession. McKeage [4] similarly pointed out that self-absorption, exclusion of other people, a quest for immediate gratification, and valuing material possessions over other things are defining features of materialism. In a review study on the relationship between materialism and well-being, Dittmar et al. [5] conceived materialism as individuals’ values, beliefs, and goals about the meaning of acquiring wealth or possessions that represent status. In short, researchers commonly conceive materialism as one’s belief about the importance and centrality of material possessions in one’s life as well as pursuits for happiness.

While common sense suggests that material possession and material consumption lead to happiness, research findings do not provide strong support for this belief. Dittmar, Bond, Hurst and Kasser [5] reviewed 259 studies and drew several conclusions based on the review. First, studies generally showed that materialism was negatively related to well-being, although the strength of the relationship differed for different measures of materialism and well-being. Second, socio-demographic factors moderated the strengths of the negative relationships between these two domains. Third, poor satisfaction of psychological needs mediates the influence of materialism on well-being. Overall, researchers concluded that “a clear, consistent negative association between a broad array of types of personal well-being and people’s belief in and prioritization of materialistic pursuits in life” was found, and “the results of this meta-analysis suggest that the negative association is robust over a number of demographic, participant, and cultural factors” [5] (p. 915).

In another review, Kasser [6] pointed out that the scientific research on materialism has been unsystematic. With the conceptualization of materialism as “a set of values/goals with particular dynamic relations to the other aims in the human value/goal system” [6] (p. 506), he summarized several observations: a tension between materialistic values and transcendental and intrinsic values; higher priority of materialistic values is associated with higher levels of personal problems (such as physical health problems and compulsory consumption) and lower level of personal well-being; materialistic people treat others less well; effective intervention of materialism covers transcendental and intrinsic goals. Additionally, he also highlighted eight issues for future research, such as the importance of conducting longitudinal research on children.

In a pioneer study on the relationships between materialism and egocentrism and adolescent delinquency in the Chinese context, Shek et al. [7] reported that both materialism and egocentrism concurrently and longitudinally predicted adolescent delinquency. Additionally, egocentrism mediated the predictive relationship between materialism and adolescent delinquency. While this study is groundbreaking, there is a need to replicate these findings. As commented by Hensel [8], “the past decade has been marked by concerns regarding the replicability and reproducibility of published research in the social sciences” (p. 577). However, although replication is an important element of social sciences, there are voices warning that replication is highly inadequate. For example, Brandt et al. [9] pointed out that “relatively few close replication attempts are reported in psychology” (p. 217).

Conceptually, there are several issues surrounding the linkage between materialism and well-being. The first issue is the conceptualization of well-being and the related measures. In the literature, while researchers have used different measures of well-being, such as subjective well-being (e.g., life satisfaction), self-evaluation (e.g., positive self-perception), mental disorders (e.g., anxiety and depression), and health risks, antisocial behavior has seldom been used as a developmental outcome indicator, particularly in adolescents. Moreno et al. [10] conceived delinquency as “behavior which violates institutionalized expectations, that is, expectations which are shared and recognized as legitimate within a social system” (p. 45). Additionally, researchers also view delinquency (e.g., gang fighting, aggression, and the destruction of others’ property) as externalizing behavior in adolescents [11,12]. Liu [11] argued that delinquency in the form of antisocial behavior is a component of externalizing behavior that is commonly regarded as an index of child and adolescent mental health. Furthermore, there are studies showing the intimate relationships between delinquency and mental health, such as the strong linkage between depression and delinquency [13] and the stimulation of serious anxiety and depression by offending [14]. From these studies, delinquency can be seen as a reflection or proxy variable for well-being, particularly when we look at the issue from a comorbidity perspective. From the perspective of holistic health, delinquency can be regarded as a problem in the social dimension of individual health because it infringes others’ rights (e.g., stealing) and violates social norms.

Studies have shown that materialism is strongly related to impulsive behavior [15] that commonly occurs in adolescent delinquency. Studies also revealed that adolescent materialism was related to delinquent behavior [16], although the studies are not systematic. There are also findings suggesting the influence of materialism on affective and cognitive impulsivity [17]. However, some clinical studies suggest impulsivity (such as in people with ADHD) may shape compulsive buying [18]; findings also showed that compulsive buying predicted materialism [19].

Regarding the linkage between values and delinquency, Bilsky and Hermann [20] remarked, “the systematic relation between delinquency and the overall spectrum of individual values has not been a particular topic of psychological research in the past” (p. 921). Nevertheless, there are studies examining the linkage between criminal attitudes and criminal behavior. For example, Wallinius et al. [21] showed that self-serving cognitive distortions (i.e., criminal attitudes) are related to criminal behavior. Based on a cost-benefit framework, Epper et al. [22] also showed that “preferences” (risk tolerance and impatience) and self-control predicted criminal behavior.

The second conceptual issue concerns the relationship between materialism and different dimensions of “self”. Primarily, there are views suggesting that self-related constructs such as negative self-appraisal and low self-esteem are causes or concomitants of materialism [23,24]. In contrast, there are also views showing that materialism leads to change in self-related constructs (i.e., consequences), such as a reduction in positive self-appraisal and the promotion of negative self-appraisals [15]. Awanis et al. [25] also asserted that materialistic people are often self-centered individuals who prefer establishing closer relationships with wealth and possessions than with people, hence underscoring the close between materialism and egocentrism. Based on an integration of the literature, Shek et al. [26] defined egocentrism as a “preoccupation that one’s self is superior/more important than other people in terms of thoughts, feelings, power, and interests. Based on such beliefs, the individual tends to focus upon one’s own advantage to the exclusion of regard for others and lack the ability to understand other people’s feelings and perspectives” (p. 298).

In the model proposed by Donnelly, Ksendzova, Howell, Vohs and Baumeister [15], it is suggested that materialism leads to “escape from self” with “cognitive deconstruction” (narrowing of attention and focus on the present) and “impulsive behavior” in the last two stages. Shek, Li, Zhu and Shek [7] put forward two arguments on why materialism is intimately linked to egocentrism. First, seeing oneself as important and self-conceit can protect one from negative emotions created by failing to reach materialistic standards. Using the psychoanalytic language, “self-inflation” in egocentrism serves as an ego defense mechanism to manage the negative perceptions generated by materialism. Second, research findings revealed a positive relationship between materialism and egocentrism as well as narcissism [27,28]. According to Whitbourne [29], while egocentrism involves the inability to see others’ views (i.e., self-focus), narcissism means a person does not care about others’ views although they see them.

Theoretically, as very few studies have explored the mediating mechanism of the association between materialism and personal well-being, we do not know much about the mediators involved. As pointed out by Górnik-Durose, while numerous studies have revealed the negative influence of materialism on individual well-being, “mechanisms underlying this association still remain partially unexplained” [27] (p. 305). Based on the study of Shek, Li, Zhu and Shek [7], we hypothesized that the relationship between materialism and adolescent delinquency would be mediated by egocentrism. Alongside research showing the relationship between materialism and egocentrism, research has shown the positive relationship between egocentrism and delinquency [30,31]. Górnik-Durose [27] also reported that narcissism mediated the relationship between materialism and well-being.

Shek, Li, Zhu and Shek [7] also pointed out several methodological problems in this field. First, most of the studies were conducted on adults, and studies involving adolescents are relatively few. A review by Dittmar, Bond, Hurst and Kasser [5] showed that only 31 studies recruited participants aged 18 and below (i.e., 12% of the studies under review). Based on his review, Kasser [6] explicitly called for more effort to understand materialism among children. Shek [32] also highlighted the inadequacy of studies on adolescent well-being. Developmentally, with cognitive and social maturation, material possession and money are new challenges for adolescents. For example, adolescents commonly compete for the possession of famous brand products, and some forms of adolescent delinquency are also linked to their urge to earn “quick money”.

Second, regarding the assessment of materialism, Kasser [6] explicitly highlighted that “researchers use many materialism measures …. although the diversity of measurement has advantages, many studies use single-item measures or measures with unknown or questionable psychometric properties” (p. 493). Across cultures, it is important to employ validated measures to assess materialism appropriate to the particular culture. Third, there are few longitudinal studies in this field. The review by Dittmar, Bond, Hurst and Kasser [5] only identified 18 longitudinal studies (i.e., 7% of the 259 studies under review). Kasser argued that research on materialism should use “prospective, longitudinal, and experimental designs” [6] (p. 507). Fourth, Shek, Li, Zhu and Shek [7] pointed out that the sample sizes in some studies were small.

Finally, most of the studies in the field are Western studies. Shek, Li, Zhu and Shek [7] argued that we need more Chinese studies because of the large population of China. Additionally, there are two other reasons why we include the related materials. First, as the data were collected from Chinese adolescents in mainland China, we have to understand the cultural context of the study. Although China has partially followed the footsteps of Western capitalism (i.e., a free-market economy such as having stock markets and private enterprises), Chinese people are still much under the influence of Chinese cultural values, particularly those based on Confucianism, Buddhism, and Taoism. Basically, over-materialistic values are not endorsed in these traditional Chinese philosophies (and not endorsed in modern China). More importantly, these philosophical frameworks basically suggest that endorsing materialistic and egocentric values is bad for the positive development of individuals. Obviously, this constitutes an interesting “indigenous” perspective on the impact of materialism on adolescent development.

Regarding Confucianism, Low [33] compared materialism and Confucianism and identified 12 differences between them, such as cherishing material possession over people versus cherishing people over material possession. Concerning Buddhism, Pace [34] outlined three core doctrines of Buddhism (e.g., one should control desires and should not fall into the trap of material cravings and consumption) and “four immeasurables” (e.g., detachment from any ego-based desires, such as material cravings and aversions). Finally, Shin and Yang [35] highlighted three underlying principles, such as “nonaction” (wu-wei) which implies the understanding of the limitations of human power, including the power of material possession. In short, the above discussion highlights Chinese cultural beliefs on materialism that shape materialistic beliefs in Chinese people. Additionally, the above discussion suggests an “indigenous” perspective on the impact of materialism on adolescent development that over-materialistic engagement would be “undesirable” for the development of an individual.

With reference to the conceptual and methodological limitations in this field, we attempted to conduct two waves of longitudinal data to investigate the relationships between materialism, egocentrism, and delinquency, with egocentrism proposed as the mediating factor. Specifically, we asked four research questions as follows:

Research Question 1: What are the relationships between adolescent materialism and delinquency concurrently and longitudinally? According to previous findings [7], we hypothesized that materialism would positively predict delinquency at each time point and over time (Hypotheses 1a and 1b);

Research Question 2: Is adolescent materialism related to egocentrism at a single time point and over time? Based on the preceding discussion, we proposed that materialism would positively predict egocentrism at each wave and over time (Hypotheses 2a and 2b);

Research Question 3: What are the concurrent and longitudinal relationships between egocentrism and adolescent delinquency? According to past findings [7], we expected that egocentrism would positively predict delinquency concurrently and longitudinally (Hypotheses 3a and 3b);

Research Question 4: Does egocentrism mediate the relationship between materialism on delinquency? Based on the findings of Shek, Li, Zhu and Shek [7], we hypothesized that egocentrism would mediate the relationship between materialism and delinquency (Hypothesis 4).

## 2. Methods

### 2.1. Participants and Procedures

To overcome the limitation of the predominance of cross-sectional studies, we employed a short-term longitudinal design with a collection of data with a six-month interval between the two waves of data in five schools in Chengdu. There were 623 and 317 public primary and junior secondary schools, respectively, and 156 schools admitting both primary and junior secondary students in Chengdu in 2020 [36]. Among these schools, we selected five to join the project, including one elementary school, one secondary school, and three schools admitting elementary and secondary school students. All five schools consented to participate. Among the participating schools, one was located in the downtown area, two were from suburban schools in the south, and two were from suburban schools in the north.

Before data collection, we obtained ethical approval from Sichuan University and consent from the school, parents, and students. Wave 1 data were collected from December 2019 to January 2020 before the COVID-19 outbreak in Wuhan, China, and the school lockdown. Wave 2 data were collected between June 2020 and July 2020 after the resumption of schools because of the gradual stabilization of the pandemic in China. During the data collection, two well-trained research assistants were present in the classrooms. As we focused on adolescents in this paper, we utilized data collected from adolescents aged 11 years or above (N = 5690 and 4981 at Wave 1 and Wave 2, respectively). There were 4981 students who completed the questionnaire at both waves (mean Wave 1 age = 13.15, SD = 1.32) with 2566 male students (51.5%) and 2415 female students (48.5%). A total of 143 students refused to participate in the study.

During the data collection process, the research assistants and schoolteachers helped go through the questionnaires quickly to ensure students responded to all questions without looking at the students’ responses. Then the research assistants collected the completed questionnaires without the involvement of the teachers. All the data were kept confidential by the research team without revealing any personal information in the research outputs.

### 2.2. Measures

#### 2.2.1. Assessment of Materialism

Shek et al. [37] developed the “Chinese Adolescent Materialism Scale (CAMS)” with 21 items based on a conceptual model with 4 dimensions. While confirmatory factor analyses supported the 4-factor model, we removed one factor only containing two items (i.e., doublet). Further analyses showed that the 19-item measure with a 3-factor structure possessed good reliability and validity. In the first cluster, the items measure the “centrality of acquiring material possession”. Some sample items include: “I believe money is everything”; “unless I can make a lot of money, I won’t respect myself”; “the amount of money one makes is a fundamental indicator of one’s success”, and “people who own wealth own everything”. In the second cluster, the items assess the “value of material possession”. Some sample items are: “possession of money can make people happy” and “having the most updated smartphone makes one happy”. In the final cluster of items, we measure hedonistic pursuits. For example, “I believe that nothing goes well for a destitute couple” and “I will not make friends with the poor”. A 6-point Likert scale was used (1 = strongly disagree; 6 = strongly agree). The mean score of the 19 items was calculated to indicate the level of materialism. Confirmatory factor analysis showed that this scale has a good factorial structure. The CAMS scores were also significantly correlated with measures of morality, spirituality, and empathy, hence giving support to the construct validity of the measure. In the present study, the 19-item scale was reliable (Cronbach’s alphas were 0.93 and 0.94 at the two waves, respectively).

#### 2.2.2. Assessment of Egocentrism

Based on the data collected from 1658 adolescents, Shek, Yu and Siu [26] developed the 19-item scale entitled “Chinese Adolescent Egocentrism Scale (CAES)”. With the deletion of five items that did not perform well, 14 items from two domains were retained. There was support for the measure’s construct validity, factorial validity, and reliability. We used the 14-item measure in this study. The cluster on “self over others/disregard of others” has the following items: “I am loyal to my own feelings even if this may upset other people”; “it doesn’t matter how other people think”; “I agree that “every man for himself and the devil takes the hindmost”; “my own benefits are more important than the benefits of other people”; “I often feel that I am more capable than people around me”; “my feelings are more important than the feelings of other people”; “no matter what happens, I can always justify my behaviors”; “the criticisms on me are usually groundless”. For the dimension of “self-conceit”, the items are: “I am a unique person”; “I feel that fortune is always on my side”; “my views are often different from the views of other people”; “I believe that my views are superior to the views of other people”; “even though my ideas are different from others’, I would insist on mine”; “my feelings are always different from the feelings of other people”. A 6-point Likert scale was adopted (1 = strongly disagree; 6 = strongly agree). The mean score of the 14 items was calculated to represent the level of egocentrism. Confirmatory factor analysis provided support for the factorial validity of the measure; the significant correlations between CAES and measures of morality, spirituality, and empathy also provided support for the construct validity of the measure. The 14-item was reliable at both waves (Cronbach’s alphas were 0.83 and 0.84 at the two waves, respectively).

#### 2.2.3. Assessment of Delinquency

To assess delinquency, we employed a validated 12-item scale that covers delinquent behaviors in the previous year [38,39]. Some delinquent behaviors include stealing, cheating at examinations, gang fights, bullying, and harassing people. The participants responded to each item on a seven-point scale (0 = “never” and 6 = “more than ten times”). The mean score over the 12 items was used in the analyses to represent the level of delinquency. There is support for the construct validity of this measure, such as the negative correlation between this measure and the indices of prosocial behavior [40], moral competence, and spirituality [41]. The scale was reliable in the present study (Cronbach’s alphas were 0.82 and 0.80 at the two waves, respectively).

The respondents were also invited to give information on their socio-demographic background (e.g., gender, age, and ethnicity) through self-report. Compared to Shek, Li, Zhu and Shek [7], there were two changes in the control variables. First, we included ethnicity as a control variable because minorities in Sichuan might migrate from the rural area to Chengdu for work. Second, we did not include “family intactness” in the present study. In mainland China, it is difficult for children to authentically obtain accurate information about “family intactness” because some divorced parents continue to live together and conceal their divorce from children [42] (p. 199). As divorce in China is highly stigmatized [42,43], divorced couples are often regarded as “selfish and shameful” [43]. Therefore, some divorced couples tend to “keep it a secret” [42] and pretend to be “married intact” to protect their children’s healthy development by giving them a “complete family”.

### 2.3. Analysis Plan

As students’ data were nested within groups (class and schools), we examined the intra-class correlations for the variables under investigation. Results showed that the mean intra-class correlation was 0.045. Based on the recommendation of Guo [44], there is no need to conduct analyses based on linear mixed models.

A series of hierarchical multiple regression analyses was conducted to answer the research questions. This approach has commonly been used in the field [45,46]. At each wave, we examined the effect of materialism or egocentrism on delinquency by entering the control variables first, followed by materialism and/or egocentrism measures. We also performed similar analyses for the longitudinal prediction of materialism on egocentrism, delinquency, and its change over time. As Steinberg et al. [47] argued, it is appropriate to use multiple regression analyses to predict scores on a variable at Wave 2 while controlling for the corresponding scores at Wave 1. We also examined the effect size of the findings through Cohen’s *f*^2^, which is an informative and standardized measure to evaluate effect size for multiple regressions [48]. According to Cohen’s [49] recommendations, Cohen’s *f*^2^ larger than 0.02, 0.15, and 0.35 represent small, medium, and large effect sizes, respectively. Finally, PROCESS analyses were performed to assess the mediating effect of egocentrism on the prediction of materialism at Wave 1 on delinquency and its change at Wave 2 [50].

## 3. Results

### 3.1. Descriptive Statistics and Reliability Analyses

Table 1 shows the means and SD for the total sample and subsamples by gender. Regarding the ethnic groups, the Han and ethnic minorities accounted for 99.1% and 0.9% of the total population in Chengdu; and among the ethnic minorities, 83% lived in urban areas, and the remaining 17% lived in rural areas [51]. In the present study, there were 4944 (99.3%) Han and 36 (0.7%) ethnic minority students (1 student did not report the information). Thus, the ethnic composition of the present sample is generally in line with the general population in Chengdu.

Table 2 shows the mean inter-item correlation coefficients and coefficient alpha values for the measures of materialism, egocentrism, and delinquency at Wave 1 and Wave 2. The findings suggest that the three measures were internally consistent at Wave 1 and Wave 2.

### 3.2. Pearson Correlation Analyses and Change in Delinquency over Time

Table 3 shows the correlation coefficients amongst materialism, egocentrism, and delinquency at each wave and over time. Results showed that the three research variables were significantly correlated at each wave and over time. Additionally, materialism and egocentrism at Wave 1 were significantly associated with delinquency at Wave 2 (*r* = 0.238 and 0.103, respectively, *p* < 0.001). Regarding the change in materialism, egocentrism, and delinquency over the two waves, results of paired *t*-tests (N = 4981) showed that Wave 1 materialism (M = 1.92, SD = 0.92) and egocentrism (M = 2.81, SD = 0.89) were lower than Wave 2 materialism (M = 2.09, SD = 1.04; *t* = −12.53, *p* < 0.01) and egocentrism (M = 2.87, SD = 0.91; *t* = −4.52, *p* < 0.001), respectively. However, Wave 1 delinquency (M = 0.30, SD = 0.48) was higher than Wave 2 delinquency (M = 0.28, SD = 0.46; *t* = 2.66, *p* < 0.001).

### 3.3. Results of Multiple Regression Analyses

For the concurrent predictors of adolescent delinquency (Table 4), after controlling for age, gender and ethnicity, separate analyses for materialism and egocentrism showed that they were significant predictors of delinquency at Wave 1 (*β* = 0.34, *p* < 0.001, Cohen’s *f^2^* = 0.120 and *β* = 0.18, *p* < 0.001, Cohen’s *f^2^* = 0.034, respectively) and Wave 2 (*β* = 0.31, *p* < 0.001, Cohen’s *f^2^* = 0.101 and *β* = 0.14, *p* < 0.001, Cohen’s *f^2^* = 0.019, respectively).

For predictions over time (see Table 5), materialism and egocentrism at Wave 1 predicted delinquency at Wave 2 (*β* = 0.22, *p* < 0.001, Cohen’s *f^2^* = 0.048 and *β* = 0.09, *p* < 0.001, Cohen’s *f^2^* = 0.008, respectively). We also examined the influence of Wave 1 materialism and egocentrism on the change in delinquency at Wave 2 by controlling delinquency scores. Table 5 shows that materialism at Wave 1 predicted an increase in delinquency over time (*β* = 0.08, *p* < 0.001, Cohen’s *f^2^* = 0.006). While the predictive effect of egocentrism at Wave 1 on change in delinquency at Wave 2 was not found (*β* = 0.01, *p* > 0.05), egocentrism at Wave 2 predicted the change in Wave 2 delinquency (*β* = 0.09, *p* < 0.001, Cohen’s *f^2^* = 0.010) after controlling the covariates.

Results revealed that materialism significantly predicted egocentrism at Wave 1 and Wave 2. Additionally, Wave 1 materialism predicted Wave 2 egocentrism (*β* = 0.20, *p* < 0.001, Cohen’s *f^2^* = 0.040) and its change over time (*β* = 0.08, *p* < 0.001, Cohen’s *f^2^* = 0.005) after controlling the covariates.

### 3.4. Mediating Effect

Table 6 and Table 7 outline the results of PROCESS analyses with 5000 bootstrap samples. We found that egocentrism at Wave 2 mediated the prediction of materialism at Wave 1 on delinquency at Wave 2 (mediating effect = 0.020, *p* < 0.001, bias-corrected 95% confidence interval = [0.012, 0.029], see Table 6). While the findings supported Hypothesis 4, we should note that the effect size was small, and there was a direct effect from materialism at Wave 1 to delinquency at Wave 2. In Table 7, we present findings on the influence of materialism at Wave 1 on the change in delinquency at Wave 2 with the mediating role of Wave 2 egocentrism (mediating effect = 0.015, *p* < 0.001, bias-corrected 95% confidence interval = [0.007, 0.023]). The findings showed that Wave 2 egocentrism partially mediated the relationship despite the small effect size.

## 4. Discussion

The present study responded to the conceptual and methodological limitations of the existing studies on the relationships between materialism and well-being [5,6], and there are several advances. First, given that limited studies were conducted in the Chinese contexts in the field, we collected data in Sichuan, China. Second, as most of the studies in this field are based on adults, we extended the investigation to adolescents. Third, in contrast to the predominance of cross-sectional studies, we adopted a short-term longitudinal study to answer the research questions. Fourth, validated measures were employed to evaluate materialism, egocentrism, and delinquency amongst Chinese adolescents. Finally, unlike some studies where small samples were employed, a large sample was used in this study.

There are also two advances in this study. First, as adolescent delinquency has seldom been used as a measure of well-being in the existing studies [11], this study expands our understanding of the research questions concerning adolescent delinquency. Based on PsycINFO, a computer search on 6 April 2022, showed that there were only 12 citations when “materialism” and “delinquency” were used. The number dropped to 2 when “Chinese” was added to the search terms. Second, the study clarifies our understanding of the linkage between materialism and egocentrism. When we searched PsycINFO using “materialism” and “egocentrism”, our search on 6 April 2022, revealed that there were only 13 citations. Third, we examined the role of egocentrism in mediating the association between materialism and delinquency. Again, this area has not been adequately researched in the existing literature.

Regarding Research Question 1, multiple regression results showed that materialism was a significant concurrent and longitudinal predictor of adolescent delinquency as well as its change over time. While acknowledging the small effect size of the significant effect, the present finding expands the literature on the predictive relationship between materialism and adolescent delinquency [7]. In particular, the findings support the common belief that preoccupation with material possession would lead to many forms of problems, such as earning quick money.

Concerning Research Question 2, Wave 1 materialism predicted egocentrism at both waves as well as change in egocentrism over time. These findings underscore the close linkage between materialism and egocentrism and suggest that materialistic beliefs may lead to exaggeration of the importance of oneself and focus on oneself. The link between materialism and egocentrism can be understood in terms of the self-focused attributes of materialism—focusing on what one possesses and what one lacks (envious of the possessions of others) as well as not being willing to share one’s possessions with others. As there are few studies in this area, further studies should be conducted to examine how materialism is related to different dimensions of egocentrism. At the macro level, it would be interesting to explore the relationship between materialism (such as obsessive emphasis on material possession) and egocentrism at the national level.

Concerning Research Question 3 on the predictive effect of egocentrism on delinquency, the present findings are consistent with the previous findings that adolescent delinquency was related to egocentrism and lack of empathy [52]. Although egocentrism at Wave 1 did not predict the change in delinquency at Wave 2, egocentrism at Wave 2 predicted the change in delinquency at Wave 2. Hence, there is a need to further clarify this issue in the future, particularly on how self-focused tendency [14] in egocentrism is related to adolescent delinquency. Finally, for Research Question 4, the present findings supported the mediating role of egocentrism in the influence of materialism on delinquency. As pointed out by Shek, Li, Zhu and Shek [7], studies examining the mediating mechanisms of the linkage between materialism and delinquency among adolescents are limited. Hence, the present study fills this gap. Second, the findings suggest that materialism may impact adolescent delinquency directly and via the mediating effect of egocentrism.

Nevertheless, alternative interpretations may explain the associations between adolescent materialism and delinquency observed in the present study. For example, there may be other variables, such as low socioeconomic status, dysfunctional values and beliefs, and undetected mental health problems, that underly both materialism and delinquency among adolescents. Additionally, adolescent misconduct may reinforce their unhealthy orientation. For example, externalizing behavior predicted adolescents’ morality in one year [53]. Thus, it is possible that delinquency may predict materialism. Given these possibilities, more studies are needed to replicate the present findings and further explore the mechanisms underlying the relationship between materialism and adolescent delinquency.

Regarding how to prevent adolescent delinquency, the present findings suggest that it is helpful to minimize materialistic beliefs and egocentrism. One possibility is to strengthen the developmental assets in adolescents, such as promoting their spirituality (e.g., finding non-material life goals) and social awareness (e.g., sensitivity to the needs and emotions of other people), and building up positive and healthy self-identity (e.g., willingness to share with others) through positive youth development (PYD) programs [54,55,56]. Based on the PYD perspective, we argue that developing PYD attributes such as moral competence and spirituality would help young people develop positive non-materialistic values so that adolescents will not inappropriately chase material possessions. Furthermore, PYD attributes such as a healthy and positive identity and social skills would help reduce egocentrism in adolescents. In Hong Kong, the Project P.A.T.H.S. has been shown to promote positive youth development attributes, which eventually helped to reduce the intention to engage in problem behavior [57,58]. In mainland China, we also showed that Tin Ka Ping P.A.T.H.S. Project promoted positive youth development [59], which strengthened the developmental assets of the program participants.

Another contribution of the study is that we replicated the findings of Shek, Li, Zhu and Shek [7]. Basically, the two studies showed several observations. First, materialism significantly predicted delinquency at both waves and over time. Second, materialism showed the positive concurrent and longitudinal prediction of egocentrism. Third, materialism predicted the change in delinquency over time. Finally, egocentrism served as a mediating factor in the relationship between materialism and delinquency. Although there was a slight difference in the control variables (non-intact family status included in the previous study and ethnicity in the present study), as the related effect was not large, we take the view that the major findings can be regarded as generalizable across time, place and populations. Actually, with reference to the checklist proposed by Brandt, Ijzerman, Dijksterhuis, Farach, Geller, Giner-Sorolla, Grange, Perugini, Spies and van ‘t Veer [9], we can conclude that the measures, analyses, instructions, and procedures are close. Interestingly, the findings are also in line with the “indigenous” Chinese conception that materialism would be linked to the negative development of an individual.

The crisis of replication has been highlighted in the literature. Freese and Peterson remarked, “science is presently in the throes of a crisis of replication” [60] (p. 148). Stevens also pointed out that “psychology faces a replication crisis” [61] (p. 1). Basically, there are two issues surrounding replication. First, there are few replication studies in social sciences. For example, Makel et al. [62] reviewed high-impact journals in Psychology and showed that few studies in Psychology are replication studies (1.07% replication rate). Second, failure to replicate is not uncommon in social science. For instance, McShane et al. also pointed out that the problem of replication “appears particularly acute in psychology, where the failure to replicate several prominent findings” [63] (p. 99). In this study, based on a different sample at a different place with data collected at different time points, the findings are generally consistent with those reported by Shek, Li, Zhu, and Shek [7].

Of course, it is understandable that the delinquent behavior of the students may be affected during COVID-19. For example, because of the restriction of social interaction and having online schooling, the chance of engaging in some delinquent behavior (e.g., gang fighting, destroying the properties of others, trespasses) would reduce. In the present study, we actually found that delinquent behavior decreased across time. With reference to the reflections highlighted in Shek [64], there is a need to understand further how materialism and egocentrism predict delinquency during the pandemic.

Although this study replicates the findings of Shek, Li, Zhu and Shek [7], we should be aware of the limitations of this study. First, as we only collected two waves of data (i.e., short-term longitudinal study), we cannot fully understand the longitudinal associations between adolescent materialism, egocentrism, and delinquency. If resources permit, we should collect more waves of data to test the chain of constructs proposed in the available theoretical models [15]. Second, although we randomly selected schools from Sichuan, the findings may not be generalizable outside the Southwestern part of China. Third, as we collected data from adolescents only, response biases such as common method variance may provide alternative explanations of the findings. Hence, the inclusion of responses of significant others and the use of multiple research methods (e.g., mixed-methods design) would be helpful.

Fourth, although there is support for the reliability and validity of the measures of materialism and egocentrism (factorial and construct validities), there is a need to collect further data on the concurrent validity and social desirability of the measures. In addition, we used self-report delinquent behavior in the present study. In future, it would be helpful to include official statistics on adolescent crimes so that we can have a more holistic picture of this problem area. However, as some adolescent delinquent behavior may not be captured by the criminal justice system (e.g., experimental drug use and free sex), official statistics also have limitations. Furthermore, many researchers are still relying on self-report measures of delinquency in their studies of adolescent delinquency [65,66]. As argued by Thornberry and Krohn [67], “although there is much room for continued improvement, self-report data appear acceptably valid and reliable for most research purposes” (p. 33).

Fifth, besides the issue of self-report delinquency, as we asked the participants to report their delinquent behavior in the past year and the interval between the two waves was around six months, there may be an overlap in the delinquency measures at these two waves. However, as we also assessed the predictive effect of Wave 1 materialism on the change in Wave 2 delinquency, this problem may be partially minimized.

Sixth, it is noteworthy that the magnitude of the effect size surrounding the significant findings is small. Hence, we should be aware of the differences between statistical and practical significance. Although statistical significance demonstrated significant effects, these effects may not be large for practical outcomes, which should be interpreted with caution. However, we should note several points. First, the magnitude of effect sizes is usually not very high in social science research, particularly in longitudinal studies [68,69]. Second, the small effect sizes of the effect of materialism on problem behaviors were also reported in similar studies. The meta-analysis by Drummond, Sauer and Ferguson [68] based on 258 independent samples showed a relatively small mean effect size (*r* = −0.15) for the relationship between materialism and personal well-being (including risk behaviors such as tobacco, alcohol, and drug use). As Dittmar et al. suggested, the use of multifaceted measures of materialist values can help clarify the effects of different aspects of materialism orientations on personal well-being.

Nevertheless, the present findings replicated the findings reported in Shek et al. [7] and provided some innovative leads for future investigation. Alongside materialism and egocentrism, many other well-known factors are also related to adolescent delinquency, including socio-demographic factors (e.g., poverty), prosocial mentality, impulsivity, and mental health status [70,71,72]. In future studies, it would be theoretically stimulating to examine how factors in different ecological systems are related to materialism and egocentrism in shaping adolescent delinquency.

## 5. Conclusions

By replicating the findings of Shek, Li, Zhu and Shek [7], this study is a positive response to the crisis of replication and the methodological limitations of the existing studies on the relationships between materialism and well-being among adolescents [5,6]. This study identified the concurrent and longitudinal prediction of materialism on adolescent delinquency and the mediating role of egocentrism between these two variables. This study has implications for juvenile delinquency prevention and intervention by suggesting minimizing materialistic beliefs and egocentrism.

## Figures and Tables

**Table 1:** Means and SD for the total sample and subsamples by gender (W1: N = 5690; W2: N = 4981).

Measures	Total Sample		Boys		Girls	
	Mean	SD	Mean	SD	Mean	SD
W1 MT	1.95	0.93	2.01	0.99	1.89	0.86
W1 EG	2.82	0.89	2.84	0.96	2.80	0.82
W1 DE	0.31	0.50	0.36	0.60	0.25	0.36
W2 MT	2.09	1.04	2.16	1.11	2.01	0.95
W2 EG	2.87	0.91	2.90	0.99	2.84	0.81
W2 DE	0.28	0.47	0.32	0.52	0.24	0.40

W1 = Wave 1; W2 = Wave 2; MT = materialism; EG = egocentrism; DE = delinquency.

**Table 2:** Reliability of measures.

Measures	Wave 1		Wave 2	
	α	Mean Inter-Item Correlation	α	Mean Inter-Item Correlation
Materialism	0.93	0.44	0.94	0.49
Egocentrism	0.83	0.28	0.84	0.30
Delinquency	0.82	0.43	0.80	0.40

**Table 3:** Descriptive and correlational analyses (W1: N = 5690; W2: N = 4981).

Measures	Mean	SD	Correlations							
			1	2	3	4	5	6	7	8
1. Age	13.15	1.32	--							
2. Gender ^a^			0.008	--						
3. Ethnic groups ^b^			0.025	−0.007	--					
4. W1 MT	1.95	0.93	0.275 ***	−0.063 ***	0.029 *	--				
5. W1 EG	2.82	0.89	0.093 ***	−0.022	−0.009	0.418 ***	--			
6. W1 DE	0.31	0.50	0.130 ***	−0.116 ***	−0.015	0.352 ***	0.191 ***	--		
7. W2 MT	2.09	1.04	0.276 ***	−0.069 ***	0.028 *	0.564 ***	0.237 ***	0.223 ***	--	
8. W2 EG	2.87	0.91	0.111 ***	−0.034 *	−0.001	0.220 ***	0.337 ***	0.128 ***	0.376 ***	--
9. W2 DE	0.28	0.47	0.101 ***	−0.084 ***	−0.016	0.238 ***	0.103 ***	0.462 ***	0.321 ***	0.149 ***

^a^ 1 = male, 2 = female; ^b^ 1 = Han, 2 = Minorities. W1 = Wave 1; W2 = Wave 2; MT = materialism; EG = egocentrism; DE = delinquency. * *p* < 0.05; *** *p* < 0.001.

**Table 4:** Cross-sectional regression analyses for delinquency.

Model	Predictors	Delinquency (Wave 1)					Delinquency (Wave 2)				
		*β*	*t*	Cohen’s *f^2^*	R^2^ Change	F Change	*β*	*t*	Cohen’s *f^2^*	R^2^ Change	F Change
1	Age	0.13	10.08 ***	0.018	0.031	60.66 ***	0.10	7.25 ***	0.011	0.018	29.94 ***
	Gender ^a^	−0.12	−8.97 ***	0.014			−0.08	−6.04 ***	0.007		
	Ethnic groups ^b^	−0.02	−1.43	0.000			−0.02	−1.27	0.000		
2	Age	0.04	3.05 **	0.002	0.104	683.64 ***	0.02	1.11	0.000	0.090	500.90 ***
	Gender ^a^	−0.10	−7.70 ***	0.010			−0.06	−4.67 ***	0.004		
	Ethnic groups ^b^	−0.03	−2.09 *	0.001			−0.03	−1.88	0.001		
	W1 Materialism	0.34	26.15 ***	0.120							
	W2 Materialism						0.31	22.38 ***	0.101		
2	Age	0.12	8.91 ***	0.014	.031	190.55 ***	0.09	6.19 ***	0.008	0.019	95.52 ***
	Gender ^a^	−0.11	−8.80 ***	0.014			−0.08	−5.75 ***	0.007		
	Ethnic groups ^b^	−0.02	−1.30	0.000			−0.02	−1.25	0.000		
	W1 Egocentrism	0.18	13.80 ***	0.034							
	W2 Egocentrism						0.14	9.77 ***	0.019		

Note. Measures of materialism and egocentrism at Wave 1 and Wave 2 were included as predictors to predict delinquency at Wave 1 and Wave 2, respectively. ^a^ 1 = male, 2 = female; ^b^ 1 = Han, 2 = minorities. * *p* < 0.05; ** *p* < 0.01; *** *p* < 0.001.

**Table 5:** Longitudinal regression analyses for delinquency.

Model	Predictors	W2 Delinquency (W1 Delinquency Was Not Controlled)					W2 Delinquency (W1 Delinquency Was Controlled)				
		*β*	*t*	Cohen’s *f^2^*	R^2^ Change	F Change	*β*	*t*	Cohen’s *f^2^*	R^2^ Change	F Change
1	Age	0.10	7.30 ***	0.011	0.018	29.71 ***	0.04	3.11 **	0.002	0.198	1244.12 ***
	Gender ^a^	−0.08	−5.90 ***	0.007			−0.03	−2.44 *	0.001		
	Ethnic groups ^b^	−0.02	−1.27	0.000			−0.01	−0.92	0.000		
	W1 Delinquency						0.45	35.27 ***	0.253		
2	Age	0.04	2.59 **	0.001	0.045	234.81 ***	0.02	1.55	0.000	0.005	29.90 ***
	Gender ^a^	−0.07	−4.82 ***	0.005			−0.03	−2.22 *	0.001		
	Ethnic groups ^b^	−0.02	−1.55	0.000			−0.01	−1.04	0.000		
	W1 Delinquency						0.43	31.60 ***	0.203		
	W1 Materialism	0.22	15.32 ***	0.048			0.08	5.47 ***	0.006		
2	Age	0.09	6.68 ***	0.009	0.008	41.67 ***	0.04	3.02 **	0.002	0.000	1.21
	Gender ^a^	−0.08	−5.75 ***	0.007			−0.03	−2.43 *	0.001		
	Ethnic groups ^b^	−0.02	−1.15	0.000			−0.01	−0.90	0.000		
	W1 Delinquency						0.45	34.55 ***	0.243		
	W1 Egocentrism	0.09	6.46 ***	0.008			0.01	1.10	0.000		

^a^ 1 = male, 2 = female; ^b^ 1 = Han, 2 = minorities. * *p* < 0.05; ** *p* < 0.01; *** *p* < 0.001.

**Table 6:** Mediating effect of Wave 2 egocentrism (the mediator) for the effect of Wave 1 materialism on Wave 2 delinquency.

Regression Models Summary	*β*	*SE*	*t*
Total effect of Wave 1 materialism (IV) on Wave 2 delinquency (DV)	0.038	0.007	15.323 ***
Wave 1 materialism (IV) to Wave 2 egocentrism (Mediator)	0.203	0.015	13.94 ***
Wave 2 egocentrism (Mediator) to Wave 2 delinquency (DV)	0.100	0.007	7.073 ***
Direct effect of Wave 1 materialism (IV) on Wave 2 delinquency (DV)	0.202	0.007	13.724 ***
Mediating effect of Wave 2 egocentrism (Mediator)	Point estimate	Bootstrapping (BC 95% CI)	
		Lower	Upper
	0.020 ***	0.012	0.029

Note. In all analyses, control variables were statistically controlled. IV = independent variable; DV = dependent variable; BC = bias corrected; CI = confidence interval. *** *p* < 0.001.

**Table 7:** Mediating effect of Wave 2 egocentrism (the mediator) for the effect of Wave 1 materialism on Wave 2 delinquency with Wave 1 delinquency controlled.

Regression Models Summary	*β*	*SE*	*t*
Total effect of Wave 1 materialism (IV) on Wave 2 delinquency (DV)	0.021	0.007	5.468 ***
Wave 1 materialism (IV) to Wave 2 egocentrism (Mediator)	0.185	0.015	12.00 ***
Wave 2 egocentrism (Mediator) to Wave 2 delinquency (DV)	0.079	0.007	6.132 ***
Direct effect of Wave 1 materialism (IV) on Wave 2 delinquency (DV)	0.062	0.007	4.375 ***
Mediating effect of Wave 2 egocentrism (Mediator)	Point estimate	Bootstrapping (BC 95% CI)	
		Lower	Upper
	0.015 ***	0.007	0.023

Note. In all analyses, control variables were statistically controlled. IV = independent variable; DV = dependent variable; BC = bias corrected; CI = confidence interval. *** *p* < 0.001.

## Data Availability

The data presented in this study are available on request from the corresponding author. The data are not publicly available due to privacy.
